# Improving access to physical healthcare for older people in mental health settings: the ImPreSs-care qualitative study

**DOI:** 10.1093/ageing/afaf261

**Published:** 2025-09-24

**Authors:** Lucy Beishon, Bethan Hickey, Bhavisha Desai, Damodar Chari, Firoza Davies, Rachel Evley, Hari Subramaniam, Elizabeta Mukaetova-Ladinska, Gregory Maniatopoulos, Tomas J Welsh, Elizabeth L Sampson, Nilesh Sanganee, Peter Neville, Cheryl Clegg, Anthony Donovan, Tom Dening, Anto P Rajkumar, Thompson Robinson, Carolyn Tarrant

**Affiliations:** Department of Cardiovascular Sciences, University of Leicester, UK; Leicester British Heart Foundation Centre of Research Excellence, UK; Leicester National Institute for Health and Care Research Biomedical Research Centre; Department of Cardiovascular Sciences, University of Leicester, UK; Leicester British Heart Foundation Centre of Research Excellence, UK; Leicester National Institute for Health and Care Research Biomedical Research Centre; Department of Cardiovascular Sciences, University of Leicester, UK; Leicester British Heart Foundation Centre of Research Excellence, UK; Leicester National Institute for Health and Care Research Biomedical Research Centre; The Evington Centre, Leicestershire Partnership Trust, UK; Department of Cardiovascular Sciences, University of Leicester, UK; Leicester British Heart Foundation Centre of Research Excellence, UK; Leicester National Institute for Health and Care Research Biomedical Research Centre; Department of Health Sciences, University of Leicester, UK; The Evington Centre, Leicestershire Partnership Trust, UK; The Evington Centre, Leicestershire Partnership Trust, UK; School of Psychology and Visual Sciences, University of Leicester, UK; University of Leicester School of Business, University of Leicester, UK; ReMindUK, Research Institute for Brain Health, Bath, UK; Bristol Medical School, University of Bristol, Bristol, UK; Royal United Hospitals Bath NHS Foundation Trust, Bath, UK; Academic Centre for Healthy Ageing, Whipps Cross Hospital, Barts Health NHS Trust, UK; Centre for Psychiatry and Mental Health, Wolfson Institute of Population Health, Queen Mary University of London, UK; NHS Leicester, Leicestershire & Rutland Integrated Care Board; Age UK Leicester Shire & Rutland; Age UK Leicester Shire & Rutland; Age UK Leicester Shire & Rutland; Institute of Mental Health, Mental Health & Clinical Neurosciences Academic Unit, School of Medicine, University of Nottingham, UK; Institute of Mental Health, Mental Health & Clinical Neurosciences Academic Unit, School of Medicine, University of Nottingham, UK; Department of Cardiovascular Sciences, University of Leicester, UK; Leicester British Heart Foundation Centre of Research Excellence, UK; Leicester National Institute for Health and Care Research Biomedical Research Centre; Department of Health Sciences, University of Leicester, UK

**Keywords:** integrated care, service delivery, dementia, severe mental illness, older people, qualitative research

## Abstract

**Background:**

Older people with serious mental ill health have high levels of physical comorbidity. Despite this, mental health services receive limited physical health support from primary or secondary care. This study investigated the facilitators and barriers to delivering physical healthcare for older people in mental health settings.

**Methods:**

In total, 54 semi-structured interviews (REC:22/IEC08/0022) were conducted with different stakeholders [staff (*n* = 28), patients (*n* = 7), carers (*n* = 19)] across two mental health hospitals. Interviews explored the facilitators and barriers to delivering physical healthcare for older people (>65 years) receiving secondary mental healthcare (dementia and psychiatric disorders). Data were analysed thematically, underpinned by a framework of integrated care for individuals living with multimorbidity.

**Results:**

A ‘multidisciplinary approach’ was valued, particularly to identify patients for targeted physical health support. There was felt to be a loss of physical health ‘training and skills’ over time, particularly amongst nursing and medical staff. Admissions to the acute hospital were potentially avoidable through improved ‘support and availability of physical health expertise’, to provide more proactive than reactive care. Alongside improved training and support, managing advanced care planning, end of life care and polypharmacy were perceived to facilitate improved physical healthcare in mental health settings.

**Conclusions:**

Lack of senior physical health leadership (e.g. geriatrician or general practitioner) and loss of skills and confidence in managing physical health in mental health settings have led to a low threshold for admissions to the acute hospital. In particular, services should support advanced care planning and end of life care from physical causes in mental health settings.

## Key Points

- Older people with significant mental ill health experience high levels of physical health comorbidities and complexity.
- Significant barriers include services not co-located, with separate management, information, and commissioning structures.
- Staff, patients, and carers values an integrated multidisciplinary approach to prioritise patients for physical health review.
- Services should include training in physical health but with sufficient leadership and support from physical health expertise.
- Services need to focus on managing polypharmacy, end of life care, and advanced care planning from physical health causes.

## Introduction

People with severe mental illness (SMI) live 15–20 years less than the general population [1]. One of the greatest factors for this inequity remains poorly managed physical health comorbidities [1]. Despite this, physical and mental health services remain fragmented across the United Kingdom (UK), and in many other countries, with little integration between services [2, 3]. In the UK, mental and physical health services and hospitals are located separately, with distinct information technology (IT), clinical records, management, and commissioning infrastructure. Mental health liaison services to acute hospitals are funded from mental health budgets, but there is a lack of reciprocity in this from physical to mental health services. This leads to frequent transfers to acute hospitals, with loss of continuity in healthcare, and increased risk of delirium [3]. Integrated care boards are National Health Service (NHS) organisations, established to tackle the challenge of delivering integrated care by developing a joint forward plan in collaboration with system partners to meet the health needs of their population, managing the NHS budget and arranging provision of health services in their local community. Despite this, significant barriers to developing and implementing integrated services remain [4, 5].

Older people who receive secondary mental health care in the UK face significant physical health challenges compared to younger people living with SMI, with greater levels of frailty, functional and cognitive impairment, polypharmacy, and higher social care needs [3]. Furthermore, the goals and focus of care are likely to shift from preventative medicine towards quality of life and advanced care planning with increasing frailty towards the end of life. Therefore, services designed for younger people, which focus on smoking cessation, safety monitoring, and cardiovascular disease prevention, are unlikely to be relevant to the ageing SMI population. In the UK, dementia [particularly associated with behavioural and psychological complications (BPSD)], is often managed by mental health services, which contrasts to some international models of dementia care [6]. As such, the physical health needs in older people, especially those with dementia, are distinct to the SMI population, with a greater focus on advanced care planning, minimising polypharmacy, quality of life and end-of-life care, compared to optimisation of chronic health conditions, and cardiometabolic risk management in patients with SMI [6]. Two recent systematic reviews highlighted a paucity of evidence on integrated physical–mental health services, although benefits were seen to care quality and satisfaction, mood, and engagement, with reductions in length of stay and falls associated with potential cost savings [6, 7]. Only two studies conducted qualitative evaluations of integrated care models, highlighting physical health complexity and co-morbidity, polypharmacy, lack of senior medical input, and involvement of carers as key challenges [8, 9].

Therefore, the primary aim of this study was to explore the barriers and facilitators to delivering integrated physical health care to older people receiving mental healthcare. Secondly, we aimed to provide evidence to inform integrated physical–mental health services tailored to the needs of older people in the UK.

## Methods

### Setting

This was an exploratory qualitative study on the perspectives of staff, patients and carers of older people receiving mental healthcare at two mental health hospitals in England. Staff, patients, and carers were recruited by poster, email, newsletter adverts, and direct invitation from clinicians at the Leicestershire Partnership Trust (LPT), Nottinghamshire Healthcare NHS Foundation Trust (NHFT), and the Age UK Leicester Shire & Rutland Dementia and Carers Support Services between January 2023 and July 2024. A summary of the hospital services is provided in Supplementary Material 1.

### Participants

Inclusion criteria were: patients and/or their carers aged >65 years receiving inpatient or community secondary mental healthcare with at least one physical health comorbidity, and staff who provide healthcare to older people in mental health services (including those admitted to general medical wards receiving or transferred from mental health services). Patients detained under the Mental Health Act were suitable for inclusion provided the direct clinical care team felt they were well enough to participate, and this would not be detrimental to their recovery. Participants were sampled purposively to maximise representation across diagnosis (e.g. SMI, dementia), setting (e.g. community, inpatient), location (e.g. Leicester, Nottingham), diversity (e.g. gender, ethnicity), and staff role (e.g. nurses, doctors, health professionals, managers, and commissioners). The study had NHS Health Research Authority approval (ref:22/IEC08/0022), and all participants provided written, informed consent, or personal consultee declaration where participants lacked capacity. The study followed the principles of the Declaration of Helsinki for medical research involving human participants.

### Theoretical framework

The Sustainable intEgrated chronic care modeLs for multimorbidity: delivery, FInancing, and performance (SELFIE) framework, a model of integrated care for people living with multimorbidity (Supplementary Table 1) [10] was used as a ‘guiding framework’ for the semi-structured interviews. The SELFIE framework encompasses six broad domains: service delivery, leadership and governance, workforce, financing, information and research, technologies and medical products, with the individual and their environment placed at the core [10]. The framework aims to understand the individual’s health and well-being, capabilities, self-management abilities, needs, and preferences in the context of their environment. The six broad domains encompass microlevels, which are likely to affect the way in which an individual access and experiences integrated care, particularly those with multimorbidity [10]. A topic guide was developed (Supplementary Material 1) informed by a systematic review of the literature [6], the SELFIE framework, and patient and public involvement (PPI). The topic guide was developed iteratively as interviews were conducted, but relevant topics were explored as they arose.

### Data collection

This study used a constructivist approach [11] to data collection and analysis, to understand the patient, carer, and staff perspective and experience of accessing and delivering physical healthcare in mental healthcare settings. Interviews were conducted in quiet rooms at the two mental health Trust premises, Age UK, the University of Leicester, patients’ homes, or via remote video link, depending on participant preference and availability. Interviews were audio-recorded (or via Microsoft Teams) and transcribed verbatim. Once transcribed, interviews were pseudonymised using a participant code and transferred to the University of Leicester for analysis. Key participant demographics were collected to summarise the characteristics of the sample (e.g. age, sex, location, diagnosis, staff role, ethnicity, number of physical health comorbidities, admissions to the acute hospital, and polypharmacy [≥5 medications]) (Table 1). Functional status of patient participants was determined using the Lawton Instrumental Activities of Daily Living scale [12].

**Table 1:** Demographics of staff, patients and carers recruited to the qualitative study. Data are mean (standard deviation) or number (percentage).

Demographic	Staff	Patients	Carers
n	28	7	19
Mean age (years, SD)	44.7 (9.9)	73.6 (6.2)	58.2 (14.2)
Sex Female (n, %)	16 (57.1)	3 (42.9)	13 (68.4)
Hospital Leicester (n, %) Nottingham (n, %)	19 (67.9) 9 (32.1)	6 (85.7) 1 (14.3)	13 (68.4) 6 (31.6)
Setting Inpatient (n, %) Community (n, %) Both (n, %)	14 (50.0) 5 (17.9) 9 (32.1)	6 (85.7) 1 (14.3) -	8 (42.1) 6 (31.6) 5 (26.3)
Staff role Medical (n, %) Nursing (n, %) Commissioners and managers (n, %) Occupational and physiotherapist (n, %) Speech, language and dietetics (n, %) Pharmacists (n, %)	13 (46.4) 2 (7.1) 3 (10.7) 6 (21.4) 2 (7.1) 2 (7.1)	— - — — — -	— - — — — -
Ethnicity Caucasian (n, %) South Asian (n, %) Black (n, %) Other (n, %)	10 (35.7) 8 (28.6) 5 (17.9) 5 (17.9)	7 (100.0) —- -	15 (79.0) 4 (21.1) — -
Patient diagnosis Dementia (n, %) Mental health (n, %) Both (n, %)	—- -	— 6 (85.7) 1 (14.3)	11 (57.4) 5 (26.3) 3 (15.8)

### Data analysis

Participants’ demographics are summarised as mean (standard deviation) or number (percentage) for continuous and nominal data, respectively. Data were analysed thematically as described by Braun and Clarke [13], aided by the use of NVivo (Version 13/R1/2020) for Windows. Five (~10%) interviews were initially open coded independently line-by-line by three researchers (LB, BH, BD) in NVivo, then reviewed by one researcher for consistency (LB) and by a PPI team member (FD) to provide reflexivity and to gain a patient and carer perspective to determine the themes in the data. Initial codes were grouped into larger themes under the six main domains of the SELFIE framework, to create a coding frame, which was then developed iteratively during coding (detailed further in Supplementary Material 1). Transcripts were coded by three researchers (LB, BD, BH), who each coded a subset of transcripts to this coding frame. Two coders (LB, BH) jointly refined and condensed the themes, and reviewed coding consistency. Where disagreements about the coding occurred, the two coders resolved these through discussion and consensus agreements. If needed, a third coder was used to arbitrate any disagreements not resolved through discussion. Themes were then refined further by reviewing and combining the SELFIE dimensions where significant overlap was evident. From this process we identified four main themes: individual with multimorbidity, service delivery and workforce, leadership and resources, and medicines, technologies, information and organisational barriers. Data extracts were exported from NVivo to Microsoft Excel to compare and contrast data within each of the over-arching themes. Data were grouped into cases in NVivo, and findings under each theme compared and summarised narratively by participant type (staff, patient, carer), diagnosis (SMI, dementia), and location (LPT, NHFT).

## Results

### Demographics

**Figure 1:**
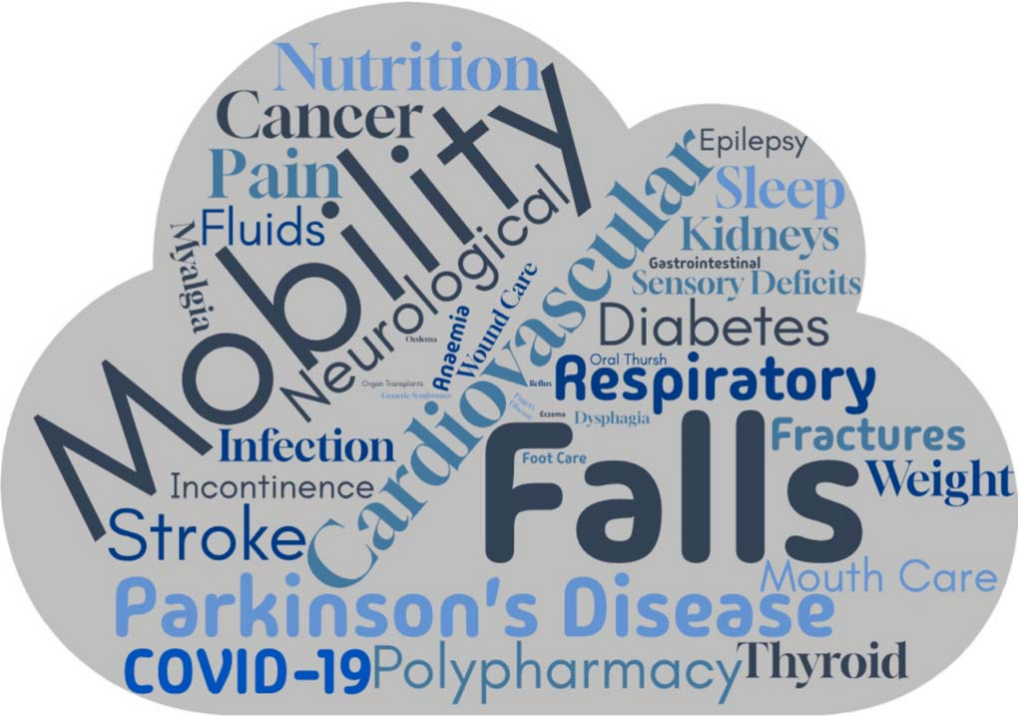
Word cloud summarising the frequency of physical health conditions coded across the interviews.

Fifty-four interviews (76% of 71 invited to participate) were conducted with staff (*n* = 28), patients (*n* = 7), and carers (*n* = 19). Patients had high levels of physical health comorbidity (median: 6.71, IQR: 6), admissions to the acute hospital (*n* = 4), polypharmacy (*n* = 7, 100%; median medications: 7, IQR: 8), and functional impairment (median Lawton IADL: 5, IQR; 4) (Table 1). The range of physical health conditions are summarised by word frequency from coding in Figure 1.

### Themes and sub-themes

**Figure 2:**
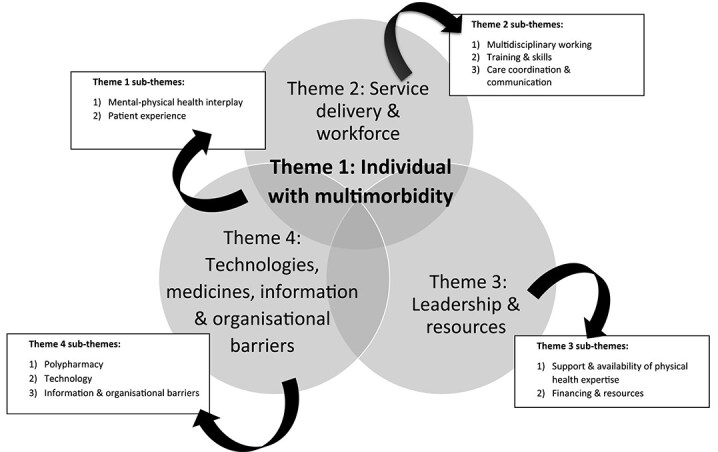
Summarising the major over-arching themes and sub-themes from the data.

The four major themes and sub-themes derived from the SELFIE framework are summarised in Figure 2 and Table 2, with additional supporting data provided in Supplementary Table 2.

**Table 2:** Themes with data from different members of multidisciplinary team, across hospitals and settings, including patients and carers. MDT = multidisciplinary team.

Themes and sub-themes	Quote
Theme 1: individual with multimorbidity	
1.1 Mental–physical health interplay	‘They affect each other a lot. If my mental health is down, my resilience is down, so I find that my pain from the fibromyalgia and my balance problems go up. I’m not sure whether they really go up, but they feel like they do. And the other way round, when there’s days I feel like I can’t get out of bed because everything’s hurting. Fibromyalgia is the one where you get pain everywhere. And if that happens then I sink into depression.’—Patient-7
1.2 Patient experience	‘How does the mental health affect the physical health and all these things. . .listening and I think giving time to the patient to let them. I’ve had so many experiences with doctors that some are so fixated on time or so fixated on just boxes and things and not adapting it to that person to get the best from the person.’—Carer-5
Theme 2: service delivery and workforce	
2.1 Multidisciplinary team working	‘Really, we should be moving away from this, one hospital for one, one hospital for another. I think learning and the education needs to be across the board from both sides. . .I think there should be better MDTs. There should be more specialist knowledge in the meetings. . .a whole MDT for the patient, looking holistically at them as opposed to mental health. . .I think that’s a whole different way of working which. . .we’re not going to achieve that in the next few years but I think it’s something that we should be working towards.’—Physiotherapist-3
2.2 Training and skills	‘So, a simple thing like changing Parkinson’s medication for a patient who is hallucinating or is having too many on/offs and those kinds of things. . .but since we aren’t an expert in that, we feel out of depth. And it’s about who takes the risk and the blame if something doesn’t work out. . .So there’s pressure from the patient, family, the legal fears and confidence in your own skills. It becomes a vicious circle. The more you rely on others, the less confident you become in your own abilities to deal with those things.’—Consultant psychiatrist-4
2.3 Care coordination and communication	‘. . .that’s the reason why we never really spoke about her physical health in the hospital just because we were completely shut down and we were told by one of the doctors that “I’m only here for her mental health and not her physical health, so if anything to do with her physical health we would have to call you in to take her to A&E”.’—Carer-2
Theme 3: Leadership and resources	
3.1 Support and availability of physical health expertise	‘. . .some of it is a cultural issue of whose job is it to look after long term or relapsing and remitting physical health problems—is it primary care?—is it specialist care?. . .so no-one’s really owning it. . .it sometimes felt that it was just being batted backwards and forwards and no-one was really owning the problem.’—Consultant Psychiatrist-2
3.2 Financing and resources	‘. . .there’s resource challenges, I mean, you are not going to just come up with somebody who can start doing this [integrated physical and mental health care]—it has to be a funded, properly commissioned and provided for service development. So that’s a challenge. A cultural adjustment and alignment need to take place so that the worlds of geriatric medicine and mental healthcare, you know, secondary care are more on the same page and not as arguably different as they are at the moment. But more than anything else, I think it’s a resource driven problem and it needs a resource driven solution.’—Consultant psychiatrist-2
Theme 4: medicines, technologies, information and organisational barriers	
4.1 Polypharmacy	‘we realised that there were a lot of drugs he was on that were interacting, so he was on medications put on by the psychiatry department and they weren’t looking at the physical medications he was on. . ..and then the hospital and the GP that were doing the pain relieving side of his medications and to treat his physical side weren’t actually looking at the psychiatric side, so we have got a lot of interactions, we had four very serious interactions going on. . .so we have got a lot of things that shouldn’t have been mixed together and no-one monitoring them.’—Carer-1
4.2 Technology	‘I’m not a big fan of video calls to be honest for my patients, especially for older patients, I’ve got a few issues with that during my previous rotation, I used to have a couple of appointments for video calls for older patients, but I feel like we’re causing more frustration to them to be honest, especially with people with sensory impairment who don’t know how to make a video call and even if they did, sometimes the quality of voice is not great and sometimes I need to just see them physically. . .I need to examine them face-to-face. So I feel like a video call for older patients is not a great option.’—Psychiatry resident doctor-2
4.3 Information and organisational barriers	‘And I think we’re not set up really for it yet. I don’t think we’re—all these, like I say wonderful ideas, are amazing, but I think still we’re different organisations. We’re different buildings. Different levels of knowledge. Different training, all the way down the scale from high up managerial level, right down to porters are differently managed. And I think we’ve got a lot of work to do to improve that. . .I think it is getting better but it’s going to be a long, long process.’—Physiotherapist-3

### Theme 1: individual with multimorbidity

#### Mental–physical health interplay and complexity

Provision of physical care in mental health settings was felt to be challenging due to the complexity and range of co-occurring mental and physical health conditions (summarised in Figure 1). Furthermore, patients, carers, and staff identified that mental and physical health interact with one another, often decompensating or exacerbating existing conditions. All participants identified high levels of physical health comorbidity and polypharmacy as problematic. This requires high levels of physical as well as mental health expertise to be able to manage the array of both physical and mental health problems experienced by patients:

‘I think they hugely impact on one another, particularly if the physical illness is one that creates pain, because I think the pain then really impacts on the depression and then the depression impacts on the movements and it’s a vicious circle really of trying to keep someone well… however, I don’t see that there’s often joined up working. That has been my experience, there’s a lack of joined up working there that causes problems.’*—Carer-1.*

#### Patient experience

As a result of the high level and complexity of physical health comorbidity a person-centred, holistic approach to integrated care is required. Levels of frailty were felt to be high amongst people with complex physical–mental health needs, and quality of life was felt to be a priority. However, patients and carers often felt there was a lack of focus on person-centred care, particularly with reference to the physical health needs, which could often be a distraction to the patient and carer priorities. This may reflect how mental health staff are sometimes uncomfortable in managing physical health needs, because of a lack of time, training, and skills in physical healthcare. Understanding how to balance competing physical health priorities for patients was seen to be particularly challenging. This was most evident in considering advanced care planning and recognising and delivering end of life care due to physical rather than mental health causes:

‘There was a lot of concern on the ward, even though she was pretty stable at the time. But I think that was just because the nursing team were like if she’s dying, what do we do? We don’t know what to do…there was a lot of worry about that and because they wanted to do the best, they couldn’t, feel it was just outside of their skill set.’*—General practitioner-1.*

### Theme 2: service delivery and workforce

#### Multidisciplinary team working

Staff working in silos was a common problem across care settings, and an MDT approach is needed to deliver high quality, integrated care. MDTs were seen to be particularly useful to identify or screen patients who would benefit from joint physical–mental health input as part of an integrated care service:

‘I think you need to have people looking with different lenses. I think it’s bringing together that kind of MDT approach for the positive outcome of the patients…and I really value that opportunity to come together…what we need more of really is those kinds of structured opportunities to come together to look who is this person in the middle of all of this? Who is this person and what are their needs encompassing the physical and mental health?’*—Speech and language therapist-1.*

#### Training and skills

A lack of training and skills in physical healthcare has reduced knowledge and expertise amongst mental health staff to support physical healthcare delivery and led to a cultural shift away from physical health provision. Mental health staff felt there has been an erosion of physical health skills and experience over time. This was particularly apparent amongst the nursing workforce who were previously dual trained but now often solely in either mental or physical health. Similarly, there has been a loss of confidence and skills amongst the medical staff working in mental health settings, with an increasing reliance on other medical specialties for support. Dedicated training and education in physical health was valued, but mental health staff felt that training and upskilling in physical health expertise should not replace adequate support from physical health clinicians. They also felt that too much emphasis on physical health practice in their work could result in dilution of their mental health expertise and skills:

‘We don’t have that kind of equipment here, we don’t have that kind of expertise here. Now to bring that to a psychiatric setting I think a) it would dilute one another’s knowledge…I think at the immediate outlook yes it would be nice having it, but I think overall it would dilute the knowledge and the skills to manage those situations’*—Mental health nurse-2.*

#### Care coordination and communication

In both inpatient and community mental health settings, patients and carers struggle to access physical healthcare, and services need to be redesigned to consider both physical–mental health needs across care settings. Whilst communication about mental health care and treatment was felt to be good with patients and carers, communication around physical healthcare needs and management was felt to be lacking. This led to a loss of trust, particularly for carers, that physical health needs were being identified and addressed appropriately. Services need to consider the role of carers, who were central to coordinating care, often managing multiple hospital appointments and visits:

‘In some cases, it needs a lot of chasing up and it’s OK for the physical health to go back to the GP but that doesn’t mean that their mental health side is kept up to date with what’s going on with them, which can make a huge difference as well. We really need the triangle of care to come into place, 100 % on there, and unfortunately the only other option you’ve got of having a triangle of care is to involve the carer themselves and let us be that sort of focal point on bringing everything together.’*—Carer-1.*

Inpatients on mental health units were particularly disadvantaged with few options to access physical health services. Staff and carers identified emergency departments as the default for accessing a physical health opinion in both care settings. When patients were not transferred to the acute hospital, mental health teams were often contacted and coordinated advice and input from multiple specialties but limited to telephone rather than face-to-face review. Unplanned hospital admissions and transfers are also logistically challenging for staff, due to managing patients detained under the mental health act, who often have challenging behavioural needs, requiring one-to-one supervision and support. Furthermore, the lack of information sharing and communication between hospitals and during transfers leads to unintended consequences for the patient’s mental and physical health provision through stopping critical medications and loss of continuity in their mental and physical healthcare treatment:

‘Often when they leave our unit, they may be on a section…we’re having staff that take them over there…It’s quite a complicated situation taking somebody to A&E…quite often medications will get stopped…They’ve no access to what our treatment goal is with the patients…that’s quite a big turmoil for the patient and for staff, because it can set people back quite considerably’*—Physiotherapist-3.*

### Theme 3: leadership and resources

#### Support and availability of physical health expertise

Lack of support and availability of senior physical health expertise has led to a need for greater physical health leadership in mental health hospitals, which often relied on junior members of staff for physical health reviews, with a reactive rather than proactive focus to physical healthcare. This was felt to lead to potentially avoidable admissions to the acute hospital by focussing on acute decompensation of physical health, rather than optimisation of chronic disease and appropriate advanced care planning:

‘…if someone with that expertise was giving us advice in a less acute way, in a more long term way, that would be really helpful for the juniors especially, and then I think for the patients as well…when you let something get to crisis point and then you have to send a patient to A&E…it’s very unsettling for the patients…and I do think if we had more regular advice maybe we’d be preventing so many back and forth admissions.’*—Psychiatry resident doctor-1.*

Geriatricians and GPs were seen as potential solutions to bridging the gap between mental and physical health services, bringing senior physical health expertise to the MDT, particularly in managing the risk and responsibility for decisions around frailty, advanced care planning, and care coordination:

‘But I don’t see secondary care mental health services liaising with secondary care physical health services very often at all actually…I think that’s a gap potentially and I think there’s something we can do to support with that…how do we embed geriatricians into our sort of mental health teams, but also thinking about it on a locality level…and with that the relationships that arise out of working together…experience then that can be shared and professional relationships that can be built up to support with the way we deliver services.’*—Commissioner-1.*

#### Financing and resources

Physical and mental health services have separate infrastructure and commissioning arrangements, and who is responsible for funding integrated care services (i.e. mental or physical health services) remains unclear. Furthermore, availability of resources remains a significant barrier to delivering integrated care, in particular the availability of primary and secondary care physicians, time, and financing. This would require a targeted approach to identifying and managing high-risk patients and bridging across care settings to maximise the resources available:

‘If there is a way to try to create a cross boundary working group to look at ways that we can do MDTs or we can have joint clinics or there is opportunity to do some more proactive case finding where visits are made to the mental health inpatient settings or there’s a regular check-in to make sure that things are understood or aware, but that will need quite a lot of significant investment both in terms of staff, money and time…but I think breaking down some of those barriers does rely on cross organisational working and trying to share our experience, knowledge, understanding, which we’re not doing at the moment.’*—Consultant geriatrician-2.*

### Theme 4: medicines, technologies, information, and organisational barriers

#### Polypharmacy

High levels of combined physical–mental health polypharmacy were particularly challenging for staff to manage in mental health settings, with patients experiencing interactions and side effects from both mental and physical health medications. Mental health teams were not confident in optimising physical health medications, meaning problematic polypharmacy was often not addressed:

‘…because we’re primarily a secondary mental health service, there’s a reluctance to change things and optimise things that aren’t mental health… But I think there’s probably not enough expertise to instil a confidence to do that…it just means there’s a more reluctant culture to think about that kind of thing…I think I could be more empowered or equipped to be thinking more holistically about medication regimes.’*—Pharmacist-1.*

#### Technology

Use of technology (e.g. remote monitoring) to support integrated care in mental health settings was limited, although this was identified as a potential opportunity to streamline or enhance face-to-face care. However, many were also sceptical about the usefulness of technology to delivering physical health care (e.g. lack of in-person examinations), particularly in older people with dementia or SMI, who may have poorer digital literacy and resources, sensory impairments, and paranoia, potentially widening inequalities in care:

‘…if they were able to engage in the technology then absolutely but for some people, I worry that these kinds of things would then introduce inequity for them and widen the gap, because those that are able to engage will and those that can’t will be deprived of that.’*—Consultant geriatrician-3.*

#### Information and organisational barriers

There were significant organisational and system level barriers to delivering integrated care, including distinct hospitals, with separate IT, clinical notes, management, and funding, and commissioning infrastructure. This means that integrated care services would often require physical health staff to work cross-site. Identifying who funds the integrated physical health service is challenging and whether this is drawn from the mental or physical health budget is a potential source of contention between services. Finally, lack of shared information systems makes provision of integrated care challenging, with physical and mental health staff unable to access, communicate, and share information across organisations:

‘…the most obvious barrier is that they’re different trusts [hospitals] on different sites…they’re not even—I mean I know at [mental health hospital] at least they're on the same site, even though they’re different trusts, whereas you have to get in a car and drive to [acute hospital] from any of the other hospitals…it’s very difficult for someone to just pop over there. And obviously with the notes not being linked up…if there was any input either way they’d need to be able to access the notes on both sides…there’s definitely lots of communication difficulties between the different trusts.’*—Consultant geriatrician-3.*

## Discussion

There are significant challenges to delivering and receiving physical healthcare for older people in both inpatient and community secondary mental health services. Levels of physical health comorbidity are high, and the complexity of managing these conditions and associated medications is increasing. There is significant interaction between physical and mental health, often destabilising each other. Services are currently reactive rather than proactive, with physical health services responding to crises, which often result in transfers to the acute hospital. Our findings highlight significant barriers to delivering integrated care such as a lack of senior physical health leadership and oversight for services, financing and resources, and the lack of co-located physical and mental health services and integrated IT systems. These could be mitigated by facilitators including provision of training and support to mental health staff, integrated MDT care with support and leadership from geriatricians and GPs, and optimisation of chronic long-term physical health and better advanced care planning for frail older people.

## Implications for practice and policy

Integrated physical care is a key priority for mental health services [2, 3, 14]. Much of the evidence base focusses on integrated care for younger people [15–17] or community services [18, 19], neglecting the different needs and challenges for older people, particularly in the inpatient setting. Older people face distinct health challenges, with greater levels of cognitive, physical and functional impairment, frailty syndromes, and higher levels of physical health comorbidity and polypharmacy [3]. Furthermore, treatment goals are likely to differ, with a greater focus on primary and secondary prevention in younger patients compared to quality of life and advanced care planning for frail older people. Here, we identified high levels of physical health need across both community and inpatient settings, but several challenges were unique to inpatients. Firstly, inpatients on mental health units have higher mental health needs, often detained under a section, with difficulty attending outpatient appointments or receiving in-person reviews. As a result, transfer to the acute hospital was often the default option for accessing a physical health opinion, but was the least preferred by patients, carers, and staff. In particular, there is significant concern around risk management and integrated services that will need to consider where the risk and responsibility for physical health management in mental health setting sits. At present, mental health staff feel under-equipped, resourced and supported to accept the risk of managing physical health in mental health settings, and mental health hospitals may be subject to greater scrutiny than acute hospitals. Hospital transfers often resulted in problematic changes to psychiatric medication, and lack of specialist mental health nursing, destabilising patients’ mental health. There was a reluctance for physical health services to review mental health in-patients, which may be due to stigma associated with patients under section in mental health services [20], as well as time and availability of staff to work cross-site. Furthermore, due to a lack of joined-up IT infrastructure, information was often lost in transfer or not handed over, leading to poor communication and care coordination between services. In the community, challenges were still evident, but patients were supported by primary care services, which is not always the case in inpatient settings. However, receiving secondary healthcare opinions remains difficult in both locations if patients are not ambulatory, and often requires coordination across a range of specialties and services, requiring significant carer support in community settings.

These challenges were similar across both mental health hospitals included in this study, although arrangements for physical health services differed. These approaches were both valued by staff, but they were not mutually exclusive, and both were important in delivering optimal physical healthcare. Geriatrician input was valued to improve care coordination, address common frailty syndromes, polypharmacy, and advance care planning for physical health. Furthermore, geriatrician input was able to bridge across care settings providing continuity in physical healthcare, which is often lacking after a long inpatient mental health stay. This was reflected in the lack of support, leadership and oversight for physical health expertise in mental health settings. This was particularly acute for resident doctors on inpatient mental health units, who often felt unsupported in managing complex physical health comorbidity. Although training and upskilling in physical health expertise was an important component of integrated care, this should not be at the expense of adequate physical health leadership and support. However, this was likely to be constrained by the local availability of staff, time and resources. Furthermore, how integrated services will be funded, commissioned and managed in an integrated fashion remains unclear.

The diagnosis of the patient is also important. In this study, patients with SMI were more likely to need optimisation of their chronic physical health conditions, falls prevention, and physical activity to prevent deconditioning. Whereas those with dementia had greater needs for advanced care planning, end of life care, and de-escalation of treatment rather than primary or secondary prevention. These aspects of care are particularly challenging to deliver when these are due to physical rather than mental health causes. Therefore, service models need to provide personalised physical health support to those with different physical and mental healthcare needs.

Table 3 summarises service recommendations mapped to the relevant theme and sub-themes derived from the SELFIE framework.

**Table 3:** Integrated physical–mental health service recommendations, mapped to the relevant theme and sub-themes from the SELFIE framework and data.

Theme and sub-theme	Integrated physical–mental health service recommendations
Theme 1: individual with multimorbidity	
1.1 Mental–physical health interplay and complexity	Provision of physical health as well as mental health expertise in mental health settings
1.2 Patient experience	Integrated services should be patient-centred and prioritise quality of life, with consideration for advance care planning and end of life care provision
Theme 2: service delivery and workforce	
2.1: MDT working	MDT approach with input from both physical and mental health services
2.2: Training and skills	Formal training and educational events for mental health staff in physical health
2.3: Care coordination and communication	Involvement of patients and their carers in both their physical and mental healthcare Improved information sharing and communication across organisations, particularly during transitions of care (e.g. discharge, hospital transfers)
Theme 3: leadership and resources	
3.1: Support and availability of physical health expertise	Integrated services need senior physical health leadership and oversight, promoting a culture of physical health in mental health settings Clarity is needed on where risk and responsibility sit for physical health management in mental health settings
3.2: Financing and resources	Integrated services need distinct funding and commissioning arrangements Services need to be resource efficient, using a proactive approach to target high-risk patients to maximise staff time and availability whilst minimising costs
Theme 4: medicines, technologies, information, and organisational barriers	
4.1: Polypharmacy	Integrated services should evaluate physical and mental health polypharmacy in tandem, identifying opportunities for optimisation and minimising drug interactions
4.2: Technology	Novel technologies and services should be considered as part of integrated services, but care is needed to avoid digital exclusion of vulnerable groups
4.3: Information, research, and organisational barriers	Integrated services need a top–down approach from funding and commissioning level, with consideration for shared IT and communication systems and co-location of services or MDT working

## Findings in context

Few international models of integrated care in specialist mental health settings exist [6, 7]. Services often have limited levels of integration, not translating to benefits in outcomes such as hospital transfers, admissions, or length of stay [9, 21–23]. Services with greater care integration had more benefits for patients [23, 24]. Benefits were identified to care quality, with greater carer satisfaction, mood, engagement, and reductions in length of stay and falls [9, 23, 24]. Two studies included cost-effectiveness analyses, demonstrating potential cost savings to integrated services [9, 25]. Few studies have fully embedded physical healthcare into mental health settings, instead opting for liaison models of care which are likely to be more financially viable and less resource intensive [9, 22]. There is good evidence to support liaison psychiatry services to acute hospitals, which are embedded nationally across the UK [26], but there is currently limited evidence available to support physical health liaison to mental health services. This contrasts with the integrated MDT approach valued by staff in this study to improve communication between services and obviate the lack of integrated IT infrastructure. This was demonstrated in qualitative evaluations of two inpatient integrated care models, where benefits were identified to delirium and dementia management, as well as opportunities for shared learning and improved communication between specialties and carers [8, 9]. In keeping with our findings, a qualitative evaluation of a geriatrician liaison model to inpatient mental health units identified high levels of physical health complexity and comorbidity, with lack of senior medical input highlighted as a key challenge [9]. Senior physical health leadership and oversight is important to improve the culture, support and confidence in providing physical healthcare in mental health settings. Few examples of digital technology to provide or support physical health interventions were identified. However, digital approaches have the potential to facilitate dedicated physical health support, reducing resource need [7], but caution is needed to avoid widening care inequalities further.

## Strengths, limitations, and future directions

This was a large qualitative interview study across inpatient and community settings at two diverse mental health hospitals. We recruited a large number of carers and staff, but relatively fewer patients, from only two hospitals, which may have introduced bias. We achieved good diversity across ethnicity in the staff group, but less-so in the patient and carer groups. Future work should recruit more patient participants and maximise diversity across ethnic minorities and socioeconomic status. Although integrated care is limited nationally [3], local, unpublished models of physical health support and liaison may not have been captured by this study, and should be explored in future work. We identified several specific challenges that would benefit from more detailed exploration. These include advance care planning and end of life care, approaches to managing physical health polypharmacy, falls prevention and physical activity intervention, and the use of digital technologies to facilitate dedicated physical health support and bridge across care settings.

## Conclusions

In conclusion, there were high levels of physical health comorbidity, complexity, and polypharmacy across inpatient and community mental health settings. Mental health services for older people would benefit from dedicated support with physical healthcare. Integrated services need dedicated funding and commissioning pathways, with provision of senior physical health support and leadership, alongside training and education for mental health staff. Services need to be embedded in the MDT and provide holistic, person-centred care with consideration for common frailty syndromes, advance care planning, polypharmacy, and falls prevention. Ideally, services should bridge across care settings, providing continuity for patients. Geriatricians have a central role to play in the provision of integrated physical care in mental health settings, but creative approaches are needed to deploy this in resource-constrained settings.

## Supplementary Material

aa-25-0569-File002_afaf261

## Data Availability

Extensive supporting data is provided in the Supplementary Material, but further data is available on request from the corresponding author.
