# Supplementary material for: Improving access to physical healthcare for older people in mental health settings: the ImPreSs-care qualitative study
Source: Age Ageing. 2025 Sep 24;54(9):afaf261. doi: 10.1093/ageing/afaf261 (PMC12459243; doi:10.1093/ageing/afaf261)
Supplement: aa-25-0569-File002_afaf261 [file aa-25-0569-file002_afaf261.docx]

**Supplementary Material**

1. **Staff Topic Guide**

**Improving physical health care in older people in mental health settings: The ImPreSs-Care Study (Staff)**

**Interview Topic Guide**

**Welcome and introduction**

**Seek consent to continue and to audio-record the interview (if applicable).**

**Let them know that no personal identifiable data will be recorded and a participant number will be allocated to them**

- Do you have experience of inpatient, community or both settings?
- Can you tell me about the health conditions your patients have.
- PROBE for physical and mental needs – which physical health conditions are particularly challenging or problematic?
- To what extent do the mental and physical conditions affect each other?
- Can you tell me about the care you have delivered for your patient’s physical health conditions during their stay on the mental health inpatient ward?
- Which health professionals have been involved to provide care for their physical needs (e.g. physio, occupational therapy, dietician, doctor, nurse, pharmacist) during their stay? Can you tell me about this. Which professionals do you feel are critical to be involved in the care for these patients?
- How well do you feel the care for their physical condition has been coordinated with their mental health care, during their stay on the mental health ward? Why? What difference did this make?
- Do you have a joint physical-mental health MDT and if not is there a role for this? Why?
- To what extent have you involved the patient or their carer in decisions about their physical and mental health care? What has helped or hindered this? How can we better involve patients and carers in their care?
- Are there any barriers to delivering care for patients from different ethnic minority or cultural backgrounds? How can we address these?
- How do you think the patients ethnic background has affected the access to care and services they have received?
- Has the patients or their carers ethnic background affected the clinical care and the service they have received during the treatment period?
- Has their ethnicity affected the effects of any medications they have received or support services they received or will receive in the community upon leaving hospital?
- Any challenges/ anxieties related to managing their condition and the kind of support you feel would be helpful in their treatment journey.
- Has your ethnic background in way had an influence on the care the patient and their carers have received? How well do you feel information has been transferred between different services about your patient’s care (e.g. hospital and GP). How can we improve their care before and after discharge from hospital?
- How do you currently use technology to help manage your patient’s care? Are there any technologies you feel would be beneficial to better manage your patient’s physical and mental health together? Probe for virtual wards, video calling etc.
- Do your patients take medications for both mental and physical health conditions? What are the impacts from side effects/interactions of these medications and how do you manage them?
- Can you tell us about your experience of advanced care planning for your patients and if not do you think there are any opportunities for this?
- Can you tell us about your experience of delivering end of life care for your patients and what were the challenges in this? Were you able to access support and if not, what support would you like to have?
- Do you feel the need for integrated physical-mental health services are greatest in the inpatient or community population and why?
- What are the key organisational/service facilitators and barriers to improving integrated physical-mental health care delivery for your patients on the mental health ward?
- What do you feel would help improve care and treatment for your patient’s physical health (or would help in ensuring all of their conditions were managed effectively as a whole) during their stay on the inpatient mental health ward?
- Is there a role for targeted geriatrician support/input for these patients and if so, why? Are there any challenges to delivering this? What benefits might this have?

**Anything not covered?** Is there anything that we haven’t covered in the interview that you think we should know or think about?

**Check that they’re ok and there are no safety concerns.**

**Closing and thanks -** check that the participant is still happy for you to use all the information provided and offer the possibility to erase sections of the recording.

Thank them for their time and contribution.

1. **Patient and Carer Topic Guide**

**Improving physical health care in older people in mental health settings: The ImPreSs-Care Study**

**Interview Topic Guide (Patients and Carers)**

**Welcome and introduction**

**Seek consent to continue and to audio-record the interview (if applicable).**

**Let them know that no personal identifiable data will be recorded and a participant number will be allocated to them**

- Can you tell me about the health conditions you have
- PROBE for physical and mental needs
- To what extent do the mental and physical conditions affect each other?
- Can you tell me about the care you have had for your physical health conditions during your stay on the mental health inpatient ward?
- Have you seen any health professionals to provide care for your physical needs (e.g. physio, occupational therapy, dietician, doctor, nurse, pharmacist) during your stay? Can you tell me about this.
- How well do you feel the care for your physical condition has been coordinated with your mental health care, during your stay on the mental health ward? Why? What difference did this make?
- To what extent have you felt involved in decisions about your physical and mental health care? What has helped or hindered this? How can we better involvement patients and carers in their care?
- How well do you feel information has been transferred between different services about your care (e.g. hospital and GP). How can we improve your care before and after discharge from hospital?
- How do you currently use technology to manage your care? Are there any technologies you feel would be beneficial to better manage your physical and mental health?
- Do you take medications for both mental and physical health conditions? What are the side effects/interactions and how do you manage them?
- What do you feel would help improve care and treatment for your physical health (or would help in ensuring all your conditions were managed effectively as a whole) during your stay on the inpatient mental health ward?
- Patients and carers from ethnic minority backgrounds: are there any additional barriers you have faced accessing physical healthcare due to your ethnicity, language or culture? What could be done to address this?
- How do you think your ethnic background has affected the access to care and services you have received?
- Has your ethnic background affected the clinical care and the service you have received during the treatment period?
- Has your ethnicity affected the effects of any medications you have received or support services you received or will receive in the community upon leaving hospital?
- Any challenges/ anxieties related to managing your condition and the kind of support you feel would be helpful in your treatment journey.

**Anything not covered?** Is there anything that we haven’t covered in the interview that you think we should know or think about?

**Check that they’re ok and there are no safety concerns.**

**Closing and thanks -** check that the participant is still happy for you to use all the information provided and offer the possibility to erase sections of the recording.

Thank them for their time and contribution.

1. **Supplementary methods**

**Structure of the hospital services**

LPT and NHFT provide inpatient, outpatient, and community mental health services across inner-city and rural locations. Neither Trust has dedicated support from an acute hospital or geriatrician (only ad-hoc telephone advice available), although one has a dedicated primary care service, whereas the other has dedicated physical health nursing support. Both hospital Trusts have access to basic blood tests and radiological imaging (e.g., chest X-rays), but limited access to more advanced imaging (e.g., computed tomography [CT] and magnetic resonance imaging [MRI]), and no access to point of care testing. Therefore, patients who require an in-person physical health review, or more detailed/rapid investigations require a transfer to the emergency department at the nearest acute hospital. Medical psychiatric units in acute hospitals are not common in the UK, although there are a few isolated examples, and only one published example[1].

**Development of the coding framework**

The initial coding framework used all six domains of the SELFIE framework. However, during refinement of the themes coded to the framework we found significant overlap between the over-arching themes. For example, service delivery and workforce had significant overlap in the data coded to them which was represented across the three sub-themes (MDT working, training and skills, care coordination and communication). As such, the broad themes were combined, with the key sub-themes represented under this broader over-arching theme. The resulting themes are grounded in, and represent the best fit to, our data. For example, many of the barriers to service delivery and workforce focussed on how individuals work together as part of the MDT, as well as communication and coordination of care between the MDT members. Similarly, the ability to provide senior oversight and leadership was closely linked to the availability of staff time and resources, which were often limited by financing. We did not identify much data coded to research and as such we have not presented data on this domain. Information and organisational barriers were combined as much of the data coded to this theme referred to a lack of integration between IT systems and infrastructure of the different hospitals, as well as a lack of sharing of communication between organisations. This referred to the higher-level organisation and system level barriers rather than the day-to-day resourcing and financing of the front-line services which focussed primarily on staff availability and time. The resulting themes are grounded in, and represent the best fit to, our data.

| **LEVEL** | **SERVICE DELIVERY** | **LEADERSHIP & GOVERNANCE** | **WORKFORCE** | **FINANCING** | **TECHNOLOGIES & MEDICAL PRODUCTS** | **INFORMATION & RESEARCH** |
| --- | --- | --- | --- | --- | --- | --- |
| **MICRO** | - Person-centred care - Tailored - Self-management - Informal caregiver involvement - Pro-active - Continuity - Treatment interaction | - Shared decision-making - Individualised care planning - Coordination tailored to complexity | - Multidisciplinary team - Named coordinator - Core group | - Coverage - Reimbursement - Out of pocket costs - Financial incentives | - Remote monitoring - E-health tools - Assistive technologies - EMR’s and patient portals | - Individual level data - Individual risk protection |
| **MESO** | - Organisational and structural integration - Continuous quality improvement system | - Supportive leadership - Clear accountability - Performance based management - Culture of shared vision, ambition and values | - New professional roles - Informal caregiver support - Continuous professional development | - Incentives to collaborate - Risk adjustment - Shared savings - Secured budget - Business case | - Shared information systems - Interoperable systems | - Innovative research methods - Data ownership and protection - Risk stratification |
| **MACRO** | - Service availability and access - Policies to integrate care across organisations and sectors - Market regulation | - Policy and action plans on chronic diseases and multi-morbidity - Political commitment | - Educational and workforce planning - Workforce-demography match | - Financial system for health and social care - Investments in innovative care models - Equity and access | - Access to technologies and medical products - Policies fostering technological innovation | - Access to information - Policies that stimulate research in integrated care and multimorbidity - Privacy and data protection legislation |

Supplementary Table 1. The six domains and levels of the SELFIE framework.

Supplementary Table 2. Additional supporting data. A&E=Accident and Emergency, DN=district nurse, ECG=electrocardiogram, GP=general practitioner, ICB=integrated care board, IV=intravenous, MDT=multidisciplinary team, RGN=registered general nurse, RMN=registered mental health nurse, SMI=severe mental illness.

| **Theme** | **Quote** |
| --- | --- |
| **Theme 1: Individual with multimorbidity** | |
| **1.1 Mental-physical health interplay** | *“I don’t think they [GP] are really that bothered to be truthful [about their physical health]. I think if there’s any physical health, like if my knees got worse, I’d go over there and discuss it with them…I don’t really worry about the cancer to be truthful. I’m more worried about my mental health because I don’t like being like this. I don’t suffer from mental health normally, only when I’ve got cancer. I mean I went sixteen years, and I just carried on with a normal life you know, going to coffee mornings and all sorts.” – Patient 4*  *“Just having the ear of a physical health nurse, just to bounce off anything that worried me, it was just so useful. I do find that lacking when you don’t know who to ask and you can’t ask anybody here who don’t know the patients because our patients are quite mixed with mental health and physical health. You have to take the two together, you can’t separate them because the one bounces off the other. So yeah, I’ve always found having a physical health nurse would be such a good idea.” – Physiotherapist 1* |
| **1.2 Patient experience** | *“Yeah, we've had physios involved and, to be honest with you, they weren’t that great, because his physical health is so poor and they were expecting him to do things that he literally could not do, so that was quite a struggle. And they were quite poor at the communication, to be honest, I struggled to get them to understand what was going on with him.” – Carer 1*  *“So, I’d asked to see matron. It wasn’t documented and what would be put into place would be two hourly turnings and two hourly checks for him because he’s now doubly incontinent since he’s been in hospital. And clearly the incontinence pads were not as absorbent as they really should have been. And my question was, “Do you have a tissue viability or do you have continent teams that you can reach?” so I’ve been his advocate in terms of his clinical needs.” – Carer 16* |
| **Theme 2: Service delivery and workforce** | |
| **2.1 Multidisciplinary working** | *“But this is where things are very asymmetrical, because there is no team embedded in mental healthcare that provides physical health in-reach…so getting everyone in the same room or even on the same screen for a virtual meeting depends entirely on the enthusiasm and energy of the individual clinicians concerned.” – Consultant Psychiatrist 2*  *“I think the whole MDT, yeah, everybody’s business, like healthy eating and just making sure we’re offering all the support to the patient, not just focusing on the mental health side, but making sure that the physical health stays in the forefront as well because that’s, you know, you need to offer people support for both.” – Dietician 1* |
| **2.2 Training & skills** | *“To be a physiotherapist in a mental health ward for older people, you need to have been a physio for some time, you need to have experience of all the different kinds of physio that they used to do because when we get to support people at the end of their life, they have various physical health conditions….So, I think as a physiotherapist you cannot come in here as a Band 5 post-graduate because you would not have the experience to work with all of this, so I think working in mental health you need to have that wider experience.” – Physiotherapist 1*  *“I think we've got a long way to go. However, we’re quite proud of ourselves in this team that we are trying to develop what we can offer physical healthcare wise and we are building skills and sending staff on training, but there is a long way to go and we do need to link in, but I do think that with the new locality working, the transformation that’s happening within the community and that patients will be in locations and the locations will be the same for mental health as GPs and DNs and so it should all linked together. I do attend the physical health and wellbeing planning group, so I know there’s work being done there that links with the GPs and there’s a lot of work being done to link mental health and physical health and increase the understanding of each other’s roles and to work out what’s out there as well. Voluntary sector, as you say, other professionals.” – Community Mental Health Team Lead 1* |
| **2.3 Care coordination & communication** | *“The other issue with that is that it can fall between services. So, if part of that check has been carried out in a hospital based mental health setting, for example, the blood testing or part of the checks that have been undertaken, there may not be a completion of the full health check. So, there is a disjointedness sometimes that can occur in terms of who's taking responsibility, particularly if that patient is seen to be under the care of a specialist mental health service.” – Commissioner 1*  *“I think it’s kind of disjointed, no I don’t think that there’s a lot of coordination, I think we deal with physical health conditions as ad hoc so when they arise and we don’t actively seek out to treat physical health conditions unless they're kind of obvious.” – Psychiatry Registrar 1*  *“It can also mean that if they have a diagnosis or a label, then clinical staff tend to focus on that particular condition, potentially over and above some of the physical and other areas, it can also mean that they're less likely to engage with routine monitoring and routine health checks or screening because their mental health condition again tends to take priority…..and some of that depends on their access to family and carers who will support with those other sides of things as well.” – Commissioner 1* |
| **Theme 3: Leadership & resources** | |
| **3.1: Support & availability of physical health expertise** | *“You’ve got to have somebody leading the physical side, because it is like [when asking senior staff questions] “Well, I’ve not been here for a week, so I don’t know him” or “I’m part time so I don’t know that”. Why? Where’s the communication? Where is it written? You should know. If leadership is not there – and I’m not saying it’s not – you know, who’s taking the clinical, practical side of it? That clinical side is just not there. There has to be a practical, clinical component that looks and says “This person has not had his bowels open, this person’s urine is trickling off, how are we going to manage that? Do we need to do a urine, do we need to dip his urine to see whether he’s got any infection? He's sleeping more, what’s the reason?” All those sides of it have been left in my opinion, and that takes nothing away from the staff, it’s just within the mental unit setting there is a component of care where there’s a gap.” – Carer 16*  *“No, like, I wish we had – so once a week we have our [mental health trust] GP, she comes on the ward, if we had someone that would be more present throughout, I think things would be looked at in a different way. And still considering how much the physical needs of our patients have increased we need better trained staff probably to look at those things as well. I know when – there’s been a lot of shifts in management and now we have like from – so we are all kind of the nursing team – so we have like a clinical head of the team and then we have the operational that has some physical health experience and I don’t know if they're recruiting nurses they look into the physical health experience, but there’s been a lot of talk within [mental health trust] about the physical health needs, but it’s a long way to go. I guess it’s not represented the way we would want to.” – Physiotherapist 2*  *“Yeah, they have, they receive both physical and mental treatments within the ward, because they have their medication being given. I don’t think we really have enough general nurses, as in RGN, within the mental health setting in that regard. So, I think it would be more beneficial if we have general practitioners or general nurses on the ward that will be looking into their physical health needs, while alongside their mental health nurses looking after their mental health needs.” – Occupational Therapist 2* |
| **3.2: Financing & resources** | *“I think every time you get somebody that’s got dehydration and you're having to look at IV fluids…..we absolutely couldn’t give IV fluids on that ward, we didn’t have any of the equipment for it, so my experience working on the wards….was that you'd have to send them straight over……and it was the same for bladder scans, we didn’t have a bladder scanner so we would have to send them to hospital just to get their bladder scanned, when you see how simple a process it is it’s really frustrating to have to do that.” – Trusted Assessor and Mental Health Nurse 1*  *“I think a lot of that is also down to the financial challenges that we’re experiencing as well, do not ever forget that we’re always under this dark space where we just are experiencing so many financial challenges. And it is quite common knowledge now that the ICBs are really having to cut back and halt some of their expenditure because of the situation that we’re in. The councils, local public health – similar situations, so yeah, investment is required…It comes back to the point I made about challenging financial climate that we’re in – every service is now looking at sustainability – it’s looking at how do they cut the corners – cut the whatever – obviously trying to not compromise the quality of care that’s being delivered – but it’s survival of the fittest isn’t it.” – Commissioner 2* |
| **Theme 4: Medicines, technologies, information and research** | |
| **4.1 Polypharmacy** | *“Rare is the patient that you would encounter in a mental health inpatient setting who wasn’t on a very high level of polypharmacy for physical health conditions. I mean, occasionally you get someone who wasn’t on any physical healthcare drugs and you think what’s wrong, you know, is this patient’s medication not been brought in, what’s been missed? So, it hardly ever happens, most people are on lots of drugs and most of them for physical health conditions.” – Consultant Psychiatrist 2*  *“So, I would say the consultants because they are psychiatrists, their area is psychiatry, they will be comfortable at maybe encouraging the junior doctors to deprescribe, because they can rationalise it comfortably, but I would say a lot of the physical health deprescribing, that’s left to the junior doctors.” – Pharmacist 2*  *“Complicated. Important. And pervasive. And there are lots of physical healthcare medications which have mental health side effects or may interact with mental health medications and the same is true the other way round, that patients may, particularly if they're on complicated regimes of medication as a mental health inpatient, more of the mental health medications may have very important consequences in terms of physical health conditions which may be exacerbated or physical health medications with which they may interact, so that’s a real hornet’s nest.” – Consultant Psychiatrist 2*  *“Also, the other issues around polypharmacy and these people are on pretty amazing combinations of anticholinergic burdens, high scores, and what I might deem not to be appropriate might be working for them. And so, it’s about not treading on toes and just think of holistic – it’s not always straightforward when you're giving a physical health consultation, but a very easy example would be around metabolic problems such as hyponatremia and knowing how much we can leave and how much they're prepared to be comfortable with in order to maintain stability with what is the tried and tested SSRI medication for mood disorders.” – Consultant Geriatrician 1* |
| **4.2 Technology** | *“I think if the carers could have like an app or, I know they're very busy, but at the end of the day, if they could send me a little synopsis of how she was just via the app. I do phone her two or three times a day to hear her voice. From her voice, you can tell a lot. But just things like, she's eaten or she's had a shower today or. So that I know that I don't have to keep asking [patient] these questions because she'll get bored of it.” – Carer 12*  *“I guess the difficulty with mental health inpatients is a vast majority of them are going to be inpatients because there isn’t a more appropriate setting for them to be managed in. Now whether some of those patients could still be enrolled into a virtual ward for medical reasons becomes a bit trickier and there’s a question there about governance and responsibility, who owns the overarching responsibility for that patient if they're an inpatient on two wards, they’d be an inpatient on a virtual ward as well as an inpatient on a mental health ward.” – Consultant Geriatrician 2* |
| **4.3 Information & organisational barriers** | *“In ideal world, I would say obviously we’d all share one tool, SystmOne, whatever was the most appropriate, whatever people use. But I know that’s – well it’s probably wishful thinking. I certainly think there needs to be, so for example things like our core assessment, which is very specifically mental health core assessment, that needs to be readily available to physical healthcare. We do have a portal that we can access within mental health services but again that’s often outdated. It won’t tell us if somebody’s just gone into A&E, what they’re in there for, anything like that. It’ll show a few scans. And we haven’t even got an up-to-date pain management or a drug management tool and those sorts of things should be across the board.” – Physiotherapist 3*  *“And then it’s they haven’t got the notes from the hearing clinic. They haven’t got the notes from the fall clinic. They haven’t got the notes for this. And I’m thinking you need the notes for everything to be able to assess what is needed.” – Patient 7 & Carer 11*  *“So, from a mental health inpatient point of view, the I think coordination of medical visits and reviews is obviously a bit of a logistical challenge. It’s the same when I go to care homes or patients’ own homes, is making sure that all the right people are in the right room at the same time and that includes patient and family can sometimes be quite tricky…..So I think logistics is probably the biggest challenge – if you can overcome that then it’s obviously very easy – get everyone in the same room and you can have a good conversation about what’s going on.” – Consultant Geriatrician 2*  *“Yeah, I think some of the barriers is definitely access to expert advice, because we are so removed, we’re a different trust, we’re a different building, we’re on our own in mental health, even though our site is really close to the [acute trust], we are separate to physical health, so I think having easy access to advice is a main barrier and I think that is one of the reasons why we let things go to crisis point sometimes before asking for help. Sometimes the barrier is the patients’ mental health, the nature of the mental health problems themselves, they can often leave us in a cyclical loop where you want to help with someone’s physical health but they can't see that yet so you can't really do anything about it at that time.” – Psychiatry Resident Doctor 1* |

1. Goldberg SE, Bradshaw LE, Kearney FC, Russell C, Whittamore KH, Foster PE, et al. Care in specialist medical and mental health unit compared with standard care for older people with cognitive impairment admitted to general hospital: randomised controlled trial (NIHR TEAM trial). BMJ (Clinical research ed). 2013 Jul 2;347:f4132.
